# Microneedle-Mediated Transdermal Drug Delivery for the Treatment of Multiple Skin Diseases

**DOI:** 10.3390/pharmaceutics17101281

**Published:** 2025-10-01

**Authors:** Lian Zhou, Shilong Xu, Siwen Li

**Affiliations:** Key Laboratory of Natural Medicines, Department of Biomedical Engineering, School of Engineering, China Pharmaceutical University, No. 639 Longmian Avenue, Jiangning District, Nanjing 211198, China; zlttkx2022@163.com (L.Z.); shilongxu@yeah.net (S.X.)

**Keywords:** MNs, stratum corneum, transdermal drug delivery, skin diseases, therapeutic applications

## Abstract

In recent years, microneedles (MNs), an innovative transdermal drug delivery system, have demonstrated significant advantages in treating diverse skin diseases. The stratum corneum (SC), with its ‘brick-mortar’ structure, is the main barrier to drug penetration into the skin. MNs—including solid, coated, hollow, dissolving, and hydrogel-forming types—penetrate it minimally to form temporary micro-channels, enabling efficient delivery of a wide range of therapeutic agents. These include small molecules, biologics, nanoparticles, and photosensitizers, among others. This technology has been effectively applied in the treatment of androgenetic alopecia, acne, scars, melanoma, psoriasis, atopic dermatitis, and vitiligo. By avoiding stimulation of dermal blood vessels and nerves, MNs offer low pain and high patient compliance. These advantages underscore their broad clinical potential for dermatologic therapy. Future studies must optimize material selection, drug-carrying efficiency, and scale-up production to facilitate clinical translation.

## 1. Introduction

The skin is the human body’s largest organ, with a surface area of up to 2 m^2^. As the outermost physical barrier, the skin protects against external physical, chemical, and biological stimulation and maintains internal environment homeostasis [1]. SC is located in the outermost layer of the skin tissue, where 15–20 layers of hydrophilic keratin-rich dead keratinocytes are arranged closely, and the intercellular space is filled with hydrophobic lamellar lipids. This unique “brick-mortar” structure makes it a major barrier to drug and foreign body penetration [2,3].

A transdermal drug delivery system (TDDS) delivers drugs across the skin into the systemic circulation at a controlled rate. Compared to oral and parenteral drug delivery, which cause limitations such as fluctuating plasma drug levels, pH, and digestive enzymes in the gastrointestinal tract that reduce drug bioavailability [4], TDDS provides continuous and controlled drug delivery, less pain, cost-effectiveness, and good patient compliance. The key to the functioning of TDDS is to break through the skin barrier to achieve delivery of the active drug [5].

**Figure 1:**
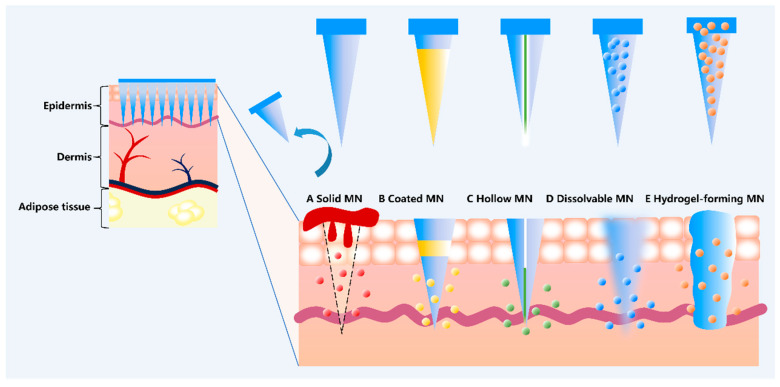
The process of drug delivery by various types of MNs.

Several strategies have been reported for breaching the SC barrier in recent decades. First-generation TDDS can only deliver small molecules of lipophilic, low-dose drugs [6]. Second-generation TDDS using chemical enhancers, noncavitating ultrasound, and iontophoresis have also been used in the clinic [7,8,9]. The third-generation TDDS utilizes MNs, thermal ablation, microdermabrasion, electroporation, and cavitation ultrasound to target the SC barrier, enabling the delivery of large molecules, including insulin and parathyroid hormone [10]. Among them, MNs consist of a series of needle-like structures arranged in an orderly fashion on the substrate, which are usually between 10 μm and 1000 μm in length, have a certain degree of mechanical strength and can penetrate the SC barrier to form an array of temporary microchannels [11,12,13]. At the same time, it avoids contact with blood vessels and nerve fibers in the deeper layers of the dermis [10], which ultimately increases the transdermal penetration of drugs in a minimally invasive manner. MNs have thus become a patient-friendly alternative to conventional drug administration techniques. Currently, MNs have been widely reported for long-term contraception [14], continuous vaccine delivery [15], oncology treatment [16], infectious disease treatment [17], and wound healing [18]. Currently, there are five main types of MNs: solid MNs, coated MNs, hollow MNs, dissolved MNs, and hydrogel MNs (Figure 1). As shown in Table 1, each has its own characteristics, advantages, and disadvantages, forming a sharp contrast between them.

Skin disease is the fourth most common human disease, affecting about one-third of the global population [19]. The most common skin diseases are alopecia, acne, scar, psoriasis, melanoma, skin infections, atopic dermatitis, and vitiligo. Drugs enter the body’s circulation via oral and injectable routes, with limited accumulation of drugs at pathological sites and unavoidable toxic side effects [20]. Surgical treatments have limitations, such as long recovery cycles and high medical costs [21], so there is an urgent need to develop novel and effective therapeutic approaches. MNs can generate reversible microchannels by inserting into the skin surface to ensure delivery of active drugs to pathological sites. More importantly, integrating these with nanoscale systems can significantly enhance their therapeutic potential. Nanocarriers such as lipid nanoparticles [22], polymeric nanoparticles [23], metal–organic frameworks [24], and extracellular vesicles [25] offer exceptional drug encapsulation efficiency, superior stability, controllable release profiles, and the ability to respond to specific pathological microenvironments—such as pH levels, enzymes, or redox gradients. By merging the physical penetration advantages of MNs with the biological targeting and responsive release capabilities of nanoscale systems, this hybrid approach enables precise and effective delivery of therapeutic agents to diseased skin sites, thereby overcoming the limitations of traditional topical and systemic administration methods. Ultimately, it can promote drug absorption [26], stimulate skin repair [27], modulate immune responses [28], and improve tissue structure [29], providing new options for the treatment of common skin diseases.

**Table 1:** Comparison of the characteristics, advantages, and disadvantages of the five types of MNs.

Classification	Characteristics	Advantages	Limitations	References
Solid MNs	Made from materials such as metal, silicon, and titanium, these devices are prepared using technologies such as laser microprocessing and form micron-level channels after piercing the SC.	High mechanical strength, reusable; Suitable for transdermal delivery of drugs applied to the surface.	Poor biocompatibility, risk of fracture, may cause infection or inflammation.	[30,31,32]
Coated MNs	The drug is coated onto the surface of the MNs in the form of a solid film using micron-level dip coating or inkjet printing technology. After insertion into the skin, the coating dissolves and releases the drug.	Easy to use, rapid drug release; Suitable for local delivery of small-molecule drugs.	Drug loading capacity is limited and depends on coating thickness and needle tip geometry; Precautions must be taken to prevent premature drug release prior to use.	[33,34,35]
Hollow MNs	The needle body has a cavity, and the needle tip has micro-holes, which can inject small volumes of solution into the dermis at a controlled rate, and can also deliver particles or nanoparticles.	Higher drug load than coated MNs; Supports liquid and particulate drug delivery, suitable for precise dosing.	After insertion, the needle tip may be blocked by dermal tissue, resulting in obstructed drug flow or inaccurate dosage.	[36,37]
Dissolving MNs	Made from biodegradable polymers (such as hyaluronic acid, chitosan, and PLGA), it degrades and releases the encapsulated drug after being inserted into the skin.	High drug loading capacity (drugs can be encapsulated in the entire needle tip matrix); Good biocompatibility and degradability, with no risk of residue.	Insufficient physical stability and mechanical strength require careful consideration during storage and use.	[38,39]
Hydrogel-forming MNs	Made from water-expandable polymers (such as gelatin and cellulose derivatives), it is inserted into the skin, where it absorbs tissue fluid and dissolves, forming a porous water microchannel for drug delivery.	Removable to avoid polymer residue; Suitable for water-soluble drugs, with a gentle drug release process.	Low mechanical strength may affect the puncture effect; similar to dissolvable MNs, stability is the main challenge.	[40,41,42]

This study followed the PRISMA guidelines and conducted a systematic literature search in the PubMed and Web of Science databases. The search strategy was constructed based on three core concepts: microneedle type, skin disease type, and treatment modality. The specific search terms are as follows:

(“microneedle”[Title/Abstract] OR “micro-needle”[Title/Abstract] OR “MN”[Title/Abstract] OR “microneedle array”[Title/Abstract] OR “dissolvable microneedle”[Title/Abstract] OR “hydrogel microneedle”[Title/Abstract]) and (“skin disease”[Title/Abstract] OR “dermatology”[Title/Abstract] OR “alopecia”[Title/Abstract] OR “acne”[Title/Abstract] OR “scar”[Title/Abstract] OR “melanoma”[Title/Abstract] OR “psoriasis”[Title/Abstract] OR “atopic dermatitis”[Title/Abstract] OR “vitiligo”[Title/Abstract]) and (“drug delivery”[Title/Abstract] OR “transdermal”[Title/Abstract] OR “topical”[Title/Abstract] OR “treatment”[Title/Abstract] OR “therapy”[Title/Abstract])

**Figure 2:**
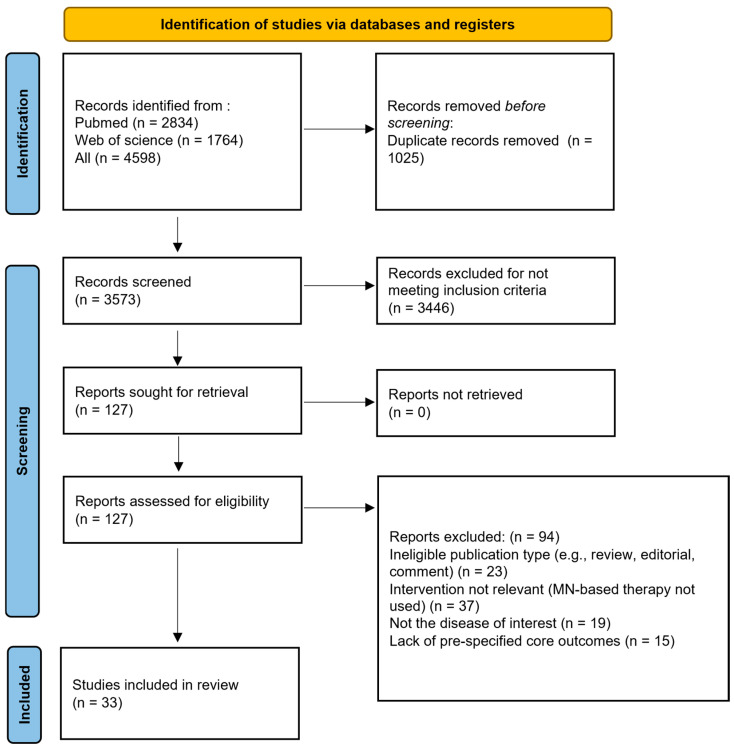
Flowchart for literature screening according to the PRISMA guidelines.

The search timeframe spans from 2021 to the present, with document types restricted to English original research articles. The search and screening flowchart is shown in Figure 2. It covers the use of MNs for drug encapsulation and innovative applications of functional MNs. It contributes to developing safer, more effective, and patient-friendly MN-based therapeutic modalities and lays the foundation for translational innovation. All abbreviations for professional terms mentioned in the text are listed in abbreviation table.

## 2. Application of MNs for Skin Disease Therapy

In recent years, skin diseases such as alopecia, acne, melanoma, psoriasis, and keloid hyperplasia have become the focus of the general public due to their obvious external symptoms and high incidence rates. Traditional systemic drug delivery techniques, such as oral and injection, are often difficult to achieve effective drug enrichment at the focal site. Although transdermal drug delivery has been widely used in treating many dermatological diseases due to its natural local targeting advantage, the efficiency of drug penetration through the skin barrier is generally poor, greatly limiting the clinical therapeutic effect. On the other hand, MN technology can form reversible micro-channels through minimally invasive methods to precisely deliver therapeutic drugs to the lesion site, which has demonstrated significant advantages and unique potential in skin disease treatment. Relevant examples are presented in Table 2.

### 2.1. MNs to Treat and Rogenetic Alopecia

Under the pathological conditions of androgenetic alopecia (AGA), the hair follicle (HF) becomes smaller, the anagen phase is shortened, the resting phase is prolonged, the length of the hair is reduced, and the terminal hairs are finally transformed into milli hairs [75]. The metabolism of androgens plays an important role in this process. Catalyzed by 5α-reductase, testosterone is irreversibly converted to dihydrotestosterone (DHT), which binds to the androgen receptor (AR), causing downstream gene regulation [76,77,78], ultimately leading to hair loss.

**Figure 3:**
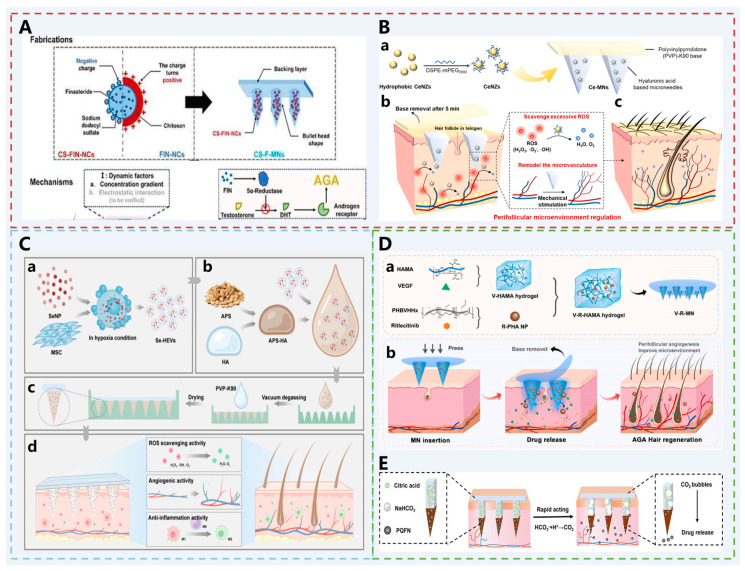
(**A**) Schematic diagram of the CS-F-MN structure and its operating principle. Reprinted with permission from ref. [43]. Copyright 2025, with permission from Elsevier. (**B-a**) The CeNZs are encapsulated in the hyaluronic acid-based MNs. (**B-b**) Ce-MNs enable direct intradermal CeNZs delivery for ROS neutralization and microvascular remodeling upon detachable backing application. (**B-c**) Ce-MNs reshape the perifollicular oxidative microenvironment and promote angiogenesis, accelerating telogen-to-anagen transition. Reprinted with permission from ref. [27]. Copyright 2021, with permission from Elsevier. (**C-a**) The descriptive preparation of Se-HEVs biological nanoparticles. (**C-b**) The composition of astragalus polysaccharide hydrogel. (C-c) The fabrication of Se-HEVs-AMN. (**C-d**) The schematic illustration of the hair growth process promoted by Se-HEVs-AMN. Reprinted with permission from ref. [44]. It is an open access article published by Elsevier. (**D-a**) Fabrication of V-R-MN. (**D-b**) The V-R-MN patch promotes hair regrowth by delivering mechanical stimulation, remodeling perifollicular vasculature via VEGF, and improving the immune microenvironment with Ritlecitinib. Reprinted with permission from ref.[45]. It is an open access article published by Elsevier. (**E**) Schematic illustration of the PQFN-loaded gas-propelled MNs for enhanced topical treatment of AGA. Reprinted with permission from ref. [46]. Copyright 2025, with permission from Elsevier.

The 5α-reductase inhibitor Finasteride (FNS) has been approved for the oral treatment of AGA [79]. However, the systemic effects of oral administration can bring about side effects that are difficult to circumvent, such as impaired reproductive function, impotence, and erectile dysfunction [80]. Zhao et al. developed a topical delivery system based on finasteride nanocrystals (FIN-NCs) modified with positively charged chitosan (CS) and combined with MNs [43] (Figure 3A). CS-FIN-NCs with a size of 200–300 nm and a positively charged surface were successfully prepared via the anti-solvent precipitation method, significantly enhancing drug accumulation in hair follicles (fluorescence intensity increased by 2.65-fold) and passive diffusion area (increased by 1.48-fold). This study pioneered the loading of CS-modified FIN-NCs onto dissolving microneedles, overcoming the skin’s stratum corneum barrier to achieve targeted drug delivery to the hair follicle region. Mechanistic studies revealed that CS-FIN-MNs improve the hair follicle microenvironment through multiple pathways: downregulating TGF-β1, upregulating β-catenin expression, and promoting angiogenesis. Ultimately, in a testosterone-induced AGA mouse model, they significantly enhanced hair follicle regeneration and hair restoration, outperforming conventional finasteride formulations.

In addition, dysregulation of the ecological niche of HF caused by excess reactive oxygen species (ROS) and insufficient vascularization in the microenvironment surrounding HF is also an important etiological factor in AGA. The expression of VEGF is decreased in HF from AGA patients, leading to a deficiency in angiogenesis, and excessive ROS can trigger premature senescence of hair papilla cells and inhibit the transformation of HF from the resting to the growing phase [81,82]. Moreover, oxidative stress and defective angiogenesis are related, and ROS accumulation has been shown to block angiogenic responses and increase vascular dysfunction.

Therefore, Yuan et al. designed a cerium dioxide nano-enzyme (CeNZ)-carrying MN patches (Ce-MNs) [27] (Figure 3B). CeNZ, with its catalase (CAT) and superoxide dismutase (SOD) activities, can alleviate oxidative stress in the pathological microenvironment of AGA. In addition to forming microchannels for CeNZ delivery, the mechanical stimulation generated by MNs can also activate the HF cycle and HF stem cells [83], trigger neovascularization, and increase the expression of genes related to hair growth [84,85]. In an established AGA mouse model, this MN patch’s efficacy was evident, with significant hair regrowth achieved on day 14. Compared to minoxidil, Ce-MNs achieved faster hair regrowth at a lower administration frequency without irreversible skin damage.

Jing et al. employed a similar principle to encapsulate selenium nanozymes (SeNPs) [44], which possess antioxidant and anti-androgenic effects, into hypoxia-pretreated extracellular vesicles (HEVs) for delivery via *Astragalus* polysaccharide (APS)-modified MNs (Figure 3C). SeNPs exhibit strong antioxidant capacity and anti-androgen effects; hypoxia pretreatment upregulates hypoxia-inducible factor 1α (HIF1α), promoting VEGF transcription and angiogenesis; APS activates the Nrf2/HO-1 pathway to inhibit inflammation and induce M2 macrophage polarization, thereby synergistically achieving antioxidant, pro-angiogenic, and anti-inflammatory effects. In an AGA mouse model, Se-HEVs-AMN demonstrated superior hair regeneration compared to minoxidil, indicating significant clinical translation potential.

In addition to excessive ROS and insufficient angiogenesis, immune inflammation is a key manifestation of hair follicle microenvironment dysregulation as well, including inflammatory responses such as perifollicular sheath thickening, mast cell degranulation, and T-cell infiltration. Ritlecitinib, a JAK3/TEC kinase inhibitor, blocks γ-chain cytokine signaling, inhibits the attack of hair follicle cells by CD8+ T cells and natural killer cells, and modulates the perifollicular immune microenvironment. Ding et al. co-loaded it with VEGF into MNs, where the two act synergistically to improve the hair follicle microenvironment [45] (Figure 3D).

Although the mechanical properties of MNs enable them to breach the skin barrier for drug delivery, transforming passive diffusion into active diffusion allows drugs to reach deeper skin layers. Based on this, Chen et al. designed a gas-driven MN system[46] (Figure 3E). They embedded sodium bicarbonate (NaHCO_3_) and citric acid in the needle base, which react upon contact with skin interstitial fluid to generate a large amount of CO_2_ bubbles. This creates a localized gas vortex field that propels the loaded puerarin/quercetin iron-chelating nanoparticles (PQFN) into the deeper dermis, thereby enabling them to more effectively clean ROS, promote angiogenesis, and reverse DHT-induced senescence of dermal papilla cells.

### 2.2. MNs to Treat Acne

Acne is a chronic inflammatory disease affecting the sebaceous glands of the HF. The global prevalence is about 9.38% [86]. Its pathogenesis is complex and involves multiple factors: (i) Androgen overexpression stimulates sebaceous glands to secrete excess sebum [87]. (ii) Abnormal follicular keratinization leads to narrowing and blockage of the follicular opening, poor sebum drainage, and formation of keratin plugs [88]. (iii) Propionibacterium acnes (*P. acnes*) breaks down triglycerides in sebum to free fatty acids, stimulating an inflammatory response in the hair follicle and surrounding tissues [89]. Severe inflammation of acne lesions often results in sequelae such as erythema, hyperpigmentation, and permanent scars, greatly impacting the patient’s life and psychology.

Antimicrobial photodynamic therapy (aPDT) is a highly selective and non-invasive treatment modality for bacterial infections [90,91]. It works by light-activated photosensitizers to generate cytotoxic ROS, which selectively oxidatively damage cellular components (lipids, proteins, and nucleic acids) to inactivate bacteria. However, aPDT has limitations such as low skin penetration, poor water solubility, and unstable free photosensitizer.

**Figure 4:**
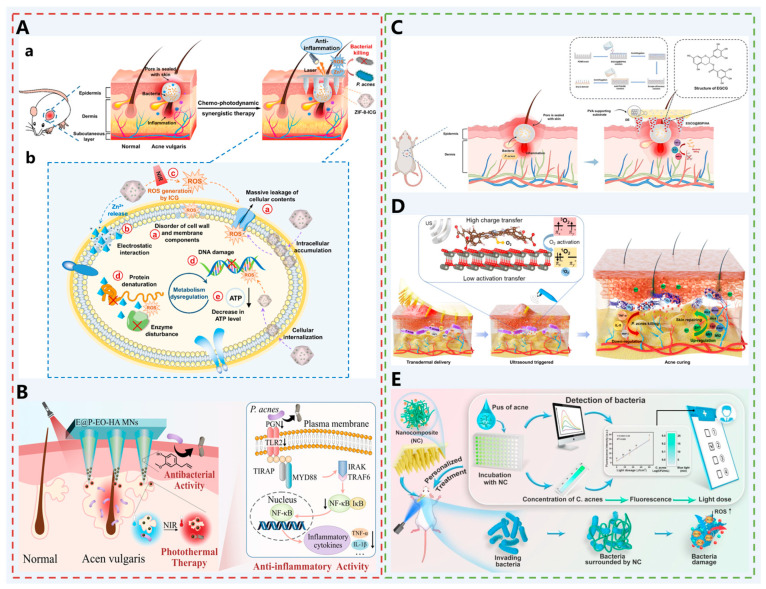
(**A-a**) Schematic illustration of the construction of multifunctional ZIF-8-ICG@MNs for amplified chemophotodynamic therapy against acne vulgaris. (**A-b**) Antimicrobial mechanisms involved in this combined strategy Reprinted with permission from ref. [47]. Copyright 2021, with permission from American Chemical Society. (**B**) E@P-EO-HA MNs promote acne healing through a synergistic action of destroying the sebaceous gland, antibacterial activity, and anti-inflammatory activity. Reprinted with permission from ref. [48]. Copyright 2024, with permission from American Chemical Society. (**C**) Schematic diagram of EGCG@BSP/HA MNs treatment for acne vulgaris. Reprinted with permission from ref. [49]. It is an open access article published by Elsevier. (**D**) Sonocatalytic mechanism and the treatment of acne through efficient sonodynamic ion therapy–based MN patch. Reprinted with permission from ref. [50]. It is an open access article published by Wiley. (**E**) Flowchart for bacterial capture and personalized photodynamic therapy of C. acnes-induced acne. Reprinted with permission from ref. [51]. Copyright 2025, with permission from Elsevier.

Therefore, Wen et al. chose biocompatible indocyanine green (ICG) as the photosensitizer and zeolite imidazolium ester framework-8 (ZIF-8) as the carrier to fabricate a drug delivery system. The carrier was formed by the self-assembly of zinc centers and 2-methylimidazole (2-MeIM) through coordination bonds, which can effectively circumvent aggregation-induced quenching of ICG and improve its photostability [47]. In addition, ZIF-8 is pH-responsive [92], which selectively degrades in the acidic microenvironment of bacterial infections, releasing Zn^2+^ and exerting a sustained antimicrobial effect. ZIF-8-ICG NP were loaded into the HA-based MNs, which was rapidly solubilized by the mesenchymal fluid, and the ZIF-8-ICG NPs were released and exposed to the 808 nm near-infrared (NIR) laser. The photosensitized ICGs produced large amounts of ROS to inhibit the overproliferation of *P. acnes* and reduce the expression of inflammatory cytokines [93]. The Zn ^2+^ was released continuously from the ZIF-8 reservoir. They disrupted the bacterial cell membrane through electrostatic interactions and increased the bacterial membrane permeability, further enhancing the therapeutic effect (Figure 4A). Ultimately, the synergistic effect of multiple perspectives was realized in treating acne.

Similarly, Wang et al. developed eugenol-loaded hyaluronic acid-dissolving MNs (E@P-EO-HA MNs) (Figure 4B) [48]. Polydopamine nanoparticles (PDA NP) are typical photothermal agents with high photothermal conversion rates, good biocompatibility, and degradability [94,95,96]. In this study, eugenol was loaded onto PDA NP (EO@PDA) and placed on the tip of HA MNs for delivery. Under 808 nm NIR light irradiation, the EO@PDA rapidly heated, destroyed sebaceous glands and inhibited the proliferation of P. acnes, which resulted in the alleviation of inflammatory responses. In addition, hydrophobic eugenol was loaded into the cavity of the MNs needle body and released when the HA MNs body was ablated, providing a continuous antibacterial and anti-inflammatory effect on the affected area and further promoting the repair of P. acnes-infected skin.

Notably, the production of pus is one of the key symptoms of acne. The primary components of pus include sebaceous gland secretions, necrotic cells, bacterial debris, and their metabolic byproducts, which collectively create an ideal environment for the proliferation of P. acnes. To address this issue, Zhang et al. developed a double-layered dissolving MN (EGCG@BSP/HA MNs) [49] (Figure 4C). The needle body comprises Bletilla striata polysaccharide (BSP), hyaluronic acid (HA), and epigallocatechin gallate (EGCG), while the base consists of PVA and diatomaceous earth (DE). The porous structure of DE enables adsorption of acne pus, preventing bacterial reinfection and providing a clean environment for the drugs loaded in the needle body. This facilitates enhanced broad-spectrum antibacterial, anti-inflammatory, antioxidant, and wound-healing functions.

Unlike traditional single-agent chemotherapy, Xiang et al. developed MNs loaded with ultrasound-responsive ZnTCPP@ZnO nanocomposites [50] (Figure 4D). Under physical ultrasound stimulation, the ZnTCPP-ZnO interface enhances electron transfer, promotes oxygen activation, and efficiently generates singlet oxygen (^1^O_2_), achieving a 99.73% clearance rate of Propionibacterium acnes within 15 min. Simultaneously, released zinc ions upregulate metallothionein and DNA replication-related genes, promoting cell proliferation and skin repair. This system also inhibits drug-resistant bacteria, achieving an integrated “physical penetration–ultrasound-catalyzed sterilization–ionic repair” treatment that offers a non-antibiotic alternative strategy for acne.

The aforementioned strategy has effectively addressed the limitations of conventional therapies (such as topical retinoids, benzoyl peroxide, oral antibiotics, etc.), including overtreatment, undertreatment, and antibiotic overuse, yet there remains room for further development, for instance, in the creation of integrated diagnostic and therapeutic strategies. Based on this, Li et al. developed a photodynamic MN (NC@MN) patch loaded with multifunctional nanocomposites (CS/CUR/TSIIA) [51] (Figure 4E). This MN efficiently captures P. acnes through positively charged chitosan (CS), utilizes the fluorescent properties of curcumin (CUR) to achieve rapid visual detection of bacterial concentration, and combines with tanshinone IIA (TSIIA) to exert anti-inflammatory effects. Based on the detected fluorescence intensity, the system can automatically match and output personalized light doses, enabling on-demand precise photodynamic therapy that effectively eliminates bacteria and alleviates inflammation, while avoiding skin damage caused by excessive ROS. This platform integrates diagnostic and therapeutic functions into a single system, achieving closed-loop management from bacterial detection to light dose regulation, and provides a new strategy for personalized treatment of acne with varying severity.

### 2.3. MNs to Treat Scar

The scar is a pathologic tissue that rises above the skin surface due to excessive collagen synthesis and insufficient degradation during wound healing after damage to the skin’s dermis, such as surgery, burns, and trauma. Wound healing is a dynamic and orderly biological process that can be divided into three successive phases: the inflammatory phase, the proliferative phase, and the remodeling phase. During the proliferative phase, new blood vessels and capillaries are generated to deliver oxygen and nutrients necessary for fibroblast proliferation. Subsequently, fibroblasts differentiate into myofibroblasts, which produce an extracellular matrix (ECM) with collagen as the main component [97]. Upon entering the remodeling phase, matrix metalloproteinases and their specific inhibitors work together to degrade the excess ECM while promoting the conversion of immature type III collagen to mature type I collagen. However, excessive angiogenesis [98] and overexpression of inflammatory mediators result in the over-differentiation of fibroblasts into myofibroblasts, creating an imbalance between collagen production and degradation, ultimately leading to scar formation.

**Figure 5:**
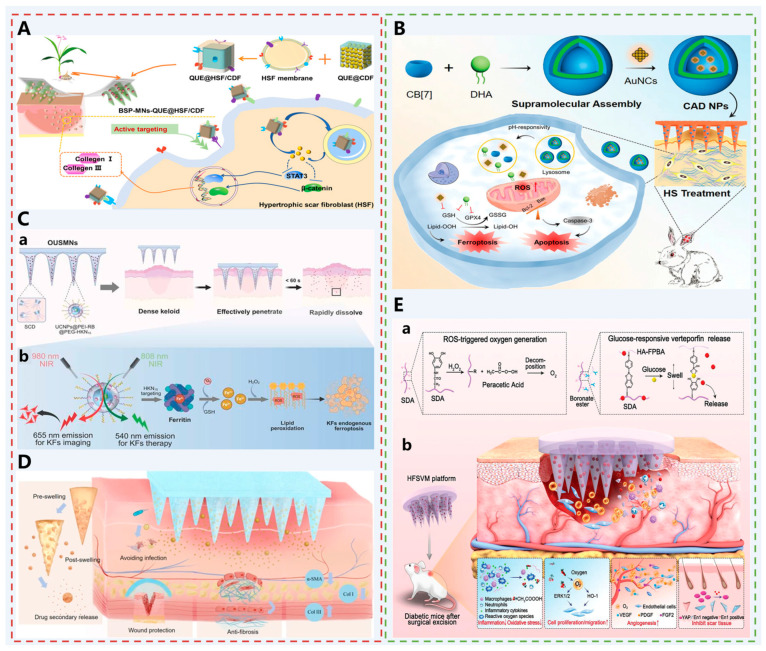
(**A**) Schematic illustration of fabrication and administration of BSP-MNs-QUE@HSF/CDF. Reprinted with permission from ref. [52]. Copyright 2021, with permission from American Chemical Society. (**B**) Schematic illustration of the self-assembled supramolecular CADNPs and their anti-scarring mechanism via concurrent ferroptosis–apoptosis pathway. Reprinted with permission from ref. [53]. Copyright 2025, with permission from John Wiley and Sons. (**C-a**) Schematic illustration of the design and application of OUSMNs in keloids. (**C-b**) The therapeutic mechanisms of UCNPs@PEI-RB@PEG-HKN_15_ for the endogenous ferroptosis and synergistic PDT in keloids. Reprinted with permission from ref. [54]. It is an open access article published by Ivyspring International. (**D**) Schematic diagram of MN-C/P-Z administration method and mechanism of action. Reprinted with permission from ref. [55]. It is an open access article published by BioMed Central. (**E-a**) HFSVM platform was fabricated by boronate ester cross-linking of SDA and HA-FPBA. (**E-b**) ROS-triggered oxygen-generating activity of SDA and glucose-responsive verteporfin release of boronate ester bonds (H_2_O_2_ is used instead of ROS). Reprinted with permission from ref. [56]. Copyright 2024, with permission from John Wiley and Sons.

Therefore, Wu et al. developed a delivery system to target hypertrophic scar fibroblasts (HSF) actively [52] (Figure 5A). The diphenylcarbonate cross-linked cyclodextrin metal–organic framework (CDF), as a novel carrier with high drug loading capacity and good biocompatibility [99,100], can be loaded with quercetin, an active drug for scar treatment. Encapsulating it within HSF membranes (QUE@HSF/CDF) enables specific targeting of HSF. Bletilla striata polysaccharide (BSP) is a naturally occurring water-soluble glucuronic acid-rich polysaccharide possessing multiple biological functions including anti-inflammatory, antioxidant, and wound-healing properties. Dispersion of QUE@HSF/CDF within BSP MNs enables delivery to the dermal layer, where it specifically targets HSF. This modulates Wnt/β-catenin and JAK2/STAT3 signaling pathways while reducing expression of type I and type III collagen in hypertrophic scars, thereby achieving effective scar treatment.

Similarly, for HSF, Zhao et al. proposed a ferroptosis–apoptosis combined therapeutic strategy for treating scar [53] (Figure 5B). Ferroptosis is a form of cell death caused by the overproduction of ROS and lipid peroxidation [101], and there is a molecular crosstalk between ferroptosis and apoptosis [102]. The combination of the two can mediate apoptosis of overproliferating fibroblasts in the pathological microenvironment of the scar. The supramolecular assembly refers to a molecular aggregate with a specific structure and function. It has the advantages of strong controllability, high drug loading rate, high bioavailability, and dynamic responsiveness [103]. Gold nanoclusters (AuNCs) can initiate apoptosis by promoting ROS production and depleting glutathione (GSH) [104,105]. Docosahexaenoic acid, the main active metabolite of dihydroartemisinin (DHA), can generate free radicals, followed by oxidative stress and induction of iron deficiency [106]. Therefore, a novel supramolecular host, cucurbituron (CB[n]s), was selected to co-assemble with AuNC and DHA to self-assemble into nanoparticles (CAD NPs). CAD NPs are pH-responsive and can exert significant antifibrotic properties by mediating an iron-apoptosis-apoptosis mechanism. It was further loaded into hydrogel MNs made of methacryloyl gelatin (GelMA) for transdermal delivery and showed significant efficacy within 3 weeks.

Although supramolecular MNs enable localized drug delivery to pathological sites, the lack of laser-responsive functionality in existing designs prevents real-time monitoring of treatment processes and on-demand therapeutic activation. This hinders closed-loop control of drug pharmacokinetics within complex pathological microenvironments. To address this, Lv et al. developed orthogonally upconverting supramolecular MNs (OUSMNs) [54] (Figure 5C). These MNs are loaded with surface-functionalized upconversion nanoparticles (UCNPs), which are modified with ferritin-targeting peptides (HKN15) and photosensitizers (rose bengal RB) to specifically target keloid fibroblasts (KFs). Under 980 nm laser excitation, UCNPs emit red light for real-time imaging and monitoring of the targeting process. At 808 nm excitation, they generate singlet oxygen (^1^O_2_), which degrades ferritin highly expressed in KFs, releasing iron ions and inducing endogenous ferroptosis. This synergizes with photodynamic therapy (PDT) to inhibit the PI3K-AKT and mTOR pathways. Combining high strength with rapid dissolution, these MNs effectively penetrate dense scar tissue and release drugs within 60 s, enabling minimally invasive, targeted, and visualizable treatment of keloids.

Although the aforementioned strategies effectively treat hypertrophic scars, implementing measures during the wound healing phase to promote scarless healing may represent a superior solution. Zhang et al. developed a composite MN system (MN-C/P-Z) combining curcumin-loaded PLGA microparticles with ZnO/GelMA hydrogel MNs [55] (Figure 5D). The MN tips loaded with curcumin-loaded PLGA microparticles (C/P) enable secondary drug release. The base layer incorporates zinc oxide nanoparticles (ZnO NPs) to form a physical barrier and exert antimicrobial effects. By penetrating the skin barrier, MNs precisely deliver curcumin deep into wounds, continuously suppressing α-SMA and collagen I expression. This inhibits fibroblast differentiation into myofibroblasts and excessive collagen deposition. ZnO NPs effectively combat Staphylococcus aureus and Escherichia coli, preventing infection and promoting a moist healing environment. These MNs exhibit excellent mechanical properties, biocompatibility, and adhesion, accelerating healing in infected wounds. By downregulating pro-fibrotic signaling pathways, they significantly reduce hypertrophic scar formation, offering a novel therapeutic strategy for achieving scar-free healing in infected wounds.

Similarly, Liu et al. developed a sericin-based ROS-responsive oxygen-releasing MN platform (HFSVM) [56] (Figure 5E). This MN is formed by cross-linking dopamine-functionalized sericin (SDA) with fluorobenzoylboronic acid-modified hyaluronic acid (HA-FPBA) via boronic acid ester bonds, and is loaded with the YAP inhibitor vitilobin. Under high ROS conditions, SDA consumes excess ROS and decomposes to generate oxygen, alleviating tissue hypoxia. It promotes angiogenesis by activating the ERK1/2 and HO-1 pathways. Hyperglycemic conditions trigger borate bond dissociation, controlling vitelinic acid release to inhibit YAP signaling and reduce En-1-positive fibroblast transformation. This synergistically regulates inflammation resolution, angiogenesis, and collagen deposition, achieving scar-free healing in diabetic wounds.

### 2.4. MNs to Treat Melanoma

Melanoma is a highly malignant tumor derived from melanocytes. Mutations of the BRAF gene and mitogen-activated protein kinase (MAPK) are common causative agents of melanoma [107]. The BRAF gene on human chromosome 7 is an important proto-oncogene encoding a serine/threonine protein kinase in the MAPK pathway, which regulates various signals within the cell. Mutations of the BRAF gene can cause BRAF phosphorylation and activation of MEK proteins (MEK 1 and MEK 2), thereby causing sustained activation of the downstream MEK-ERK signaling pathway, exacerbating melanoma growth, proliferation, invasion, and metastasis [108,109].

**Figure 6:**
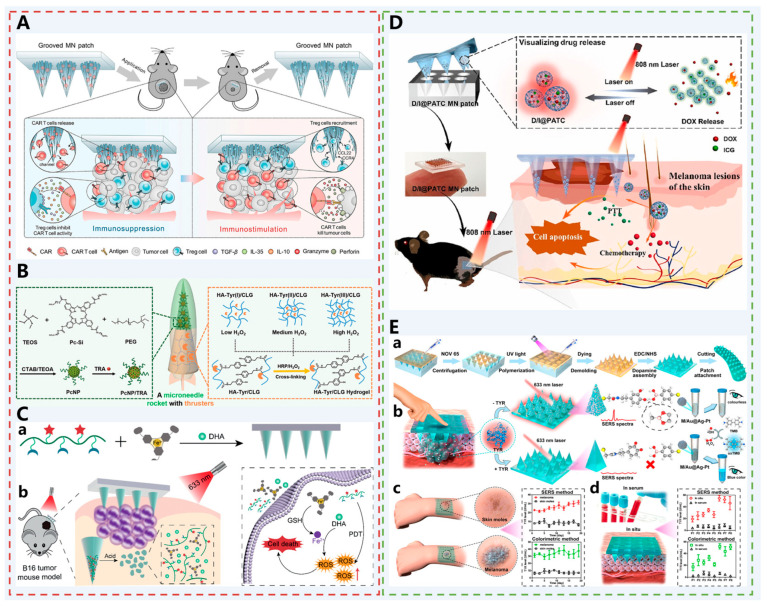
(**A**) Schematic of augmented adoptive T cell therapy using a grooved MN. Reprinted with permission from ref. [57]. Copyright 2024, with permission from John Wiley and Sons. (**B**) The structure of dually layered MN rocket with thrusters. Reprinted with permission from ref. [58]. Copyright 2024, with permission from John Wiley and Sons. (**C-a**) Schematic illustration of the fabrication of hydrogel-based DHA@HPFe-MN MNs. (**C-b**) Schematic illustration of the in vivo antitumor mechanism of DHA@HPFe-MN via oxidative stress amplification. Reprinted with permission from ref. [59]. It is an open access article published by Elsevier. (**D**) Schematic illustration of D/I@PATC MN patches for laser-triggered chemo-photothermal synergistic therapy of melanoma. Reprinted with permission from ref. [60]. It is an open access article published by Elsevier. (**E-a**) Schematic illustration of the fabricated process for the MN patch. (**E-b**) The principle of MN-based Au@Ag-Pt NPs for in situ detection of TYR. (**E-c**) Dynamic monitoring of TYR levels for screening skin moles of volunteers and melanoma patients. (**E-d**) Dual-model detection of TYR levels in the serum and skin of melanoma patients. Reprinted with permission from ref. [61]. Copyright 2023, with permission from John American Chemical Society.

Adoptive T cell therapy (ACT) is a novel immunotherapy that engineers T cells to express tumor-specific receptors, including T cell receptor (TCR) or chimeric antigen receptor (CAR), to precisely target tumor cells. [110,111,112]. However, melanomas are surrounded by abnormal ECM deposition and a dense and fibrotic microenvironment. Effector T-cell (Teff) cell infiltration is significantly reduced, and T-cell transport is greatly restricted [113]. In addition, the immunosuppressive tumor microenvironment (TME) may lead to suppression of Teff cells, e.g., regulatory T (Treg) cells greatly suppress Teff and lead to immune tolerance [112]. CCR 4, a receptor for the chemokine CCL 22, is highly expressed in Treg cells and regulates the migration of Treg cells into the TME [114,115,116]. Therefore, Zhou et al. developed a grooved MNs patch with surface-modified chemokine CCL 22, which utilized the mechanical properties of MNs to form microchannels on the skin surface for local infiltration of TCR T cells or CAR T cells into the melanoma[57]. At the same time, the surface-modified CCL 22 could efficiently attract Treg cells and divert them away from the TME (Figure 6A). The ratio of CD8+ T cells/Treg cells was thus increased, ultimately achieving tumor suppression.

Trametinib (TRA), a compound specifically targeting the MEK pathway, can be loaded into mesoporous silica nanoparticle carriers. Photosensitizers with photothermal conversion effects are known to have tumor cell-killing functions under NIR irradiation. However, free photosensitizers are unstable, and covalent attachment to mesoporous silica nanoparticles prevents aggregation and light-induced bursting. Up to this point, nanoparticles loaded with small-molecule drugs were able to synergize with PDT for the treatment of melanoma. However, the limited skin permeability of the nanoparticles limited their functionality, so they were loaded in enzyme-mediated hyaluronic acid-tyramine hydrogel (HA-Tyr/CLG)-based soluble MNs. In addition, the ECM of tumors consists of extracellular components such as collagen and proteoglycans, which severely impede the penetration and diffusion of active drugs [117]. Collagenase (CLG), which has the enzymatic activity to degrade collagen [118], can degrade and soften the ECM, loosening its structure and thus improving drug diffusion and distribution throughout the tumor mass. Therefore, Pan et al. designed a rocket-like bilayer MN structure (PcNP/TRA-HA-Tyr/CLG-MNs) [58] (Figure 6B). Its upper layer consisted of mesoporous silica nanoparticles covalently bonded with photosensitizers (PcNP/TRA) and the targeted therapeutic drug TRA, making a specific molecular pathway involved in melanoma growth. The lower layer was a hydrogel matrix encapsulating CLG with controlled cross-linking density (HA-Tyr/CLG), remodeling the tumor microenvironment and ultimately realizing the deep drug penetration and the effective combination of melanoma-targeted. The ultimate goal was to achieve deep drug penetration and effective combination therapy for melanoma.

PDT holds significant potential in melanoma treatment due to its high efficiency, non-invasiveness, and controllable spatiotemporal characteristics. However, its application is constrained by the strong hydrophobicity of photosensitizers, poor tumor selectivity, and oxygen consumption during treatment. Therefore, Huang et al. developed a pH-activated oxidative stress amplification dissolving MN system (DHA@HPFe-MN) for combined chemo-photodynamic therapy of melanoma [59] (Figure 6C). The MN tip incorporates a pH-responsive acylhydrazone-crosslinked self-assembled HA-ADH-PpIX conjugate and an “iron reservoir” PA-Fe^3+^ complex, simultaneously loaded with dihydroartemisinin (DHA) for controlled release within the acidic tumor microenvironment. Under acidic conditions, the MN degrades to release DHA and PA-Fe^3+^. The latter is reduced to Fe^2+^ by intracellular glutathione, catalyzing DHA to generate abundant ROS for oxygen-independent chemotherapy. Concurrently, under light irradiation, PpIX exerts photodynamic effects, further amplifying oxidative stress. This MN system exhibits excellent mechanical properties, skin penetration capability, and biocompatibility, enabling precise drug delivery to tumor sites. Through its pH-triggered release mechanism and oxidative stress amplification strategy, it significantly enhances therapeutic efficacy against melanoma, effectively killing tumor cells under both normoxic and hypoxic conditions. This provides a novel, intelligent delivery platform for localized combination therapy of melanoma.

Existing combined therapy systems (chemotherapy + phototherapy) demonstrate significant efficacy but remain limited by requirements for multiple dosing sessions, insufficient precision in drug release control, and the inability to directly monitor drug carrier release behavior. Therefore, Wang et al. innovatively developed a novel melanoma treatment strategy featuring single-dose precise pulsed delivery, integrated photothermal effects, and direct visual monitoring of drug release [61] (Figure 6D). The core innovation lies in introducing the thermosensitive polymer PATC, which possesses aggregation-induced emission (AIE) properties, enabling real-time visualization of the drug release process. This allows clinicians to intuitively assess whether drugs are accurately delivered to the lesion and whether the release volume is sufficient, thereby achieving precise control and feedback during treatment. Additionally, the system possesses light-controlled pulsed drug release capability, enabling multiple drug releases triggered by external light exposure from a single administration. This significantly enhances treatment compliance and controllability.

Furthermore, compared to melanoma treatment, its diagnosis and monitoring should not be overlooked. Huang et al. developed a surface-enhanced Raman scattering (SERS)-colorimetric dual-mode sensing platform based on a wearable MN patch and trimetallic Au@Ag-Pt nanoparticles for in situ monitoring of TYR activity in skin, enabling early screening and dynamic monitoring of melanoma [60] (Figure 6E). The MN patch establishes a TYR-responsive signal conversion mechanism by forming reversible borate bonds between surface-modified dopamine (containing catechol groups) and 4-mercaptobenzoic acid-labeled Au@Ag-Pt nanoparticles. In the presence of TYR, catechol is specifically oxidized to benzoquinone, inhibiting nanoparticle binding to MNs and triggering a dual-mode response: SERS signal attenuation and enhanced colorimetric signal. This overcomes limitations of traditional serum testing by directly capturing TYR activity changes at skin lesions, avoiding tissue damage and blood interference, and significantly improving early diagnostic sensitivity.

### 2.5. MNs to Treat Psoriasis

Psoriasis is a chronic inflammatory skin disease mainly characterized by the appearance of thickened red patches of skin as well as silvery scales occurring on the scalp, trunk, and extensor surfaces [119]. Its pathogenesis involves abnormal activation of the immune system and persistent inflammation. Epidermal CD8+ T cells express key disease mediators IL-17 and IL-22, both of which induce hyperproliferation of keratinocytes through activation of Th17 in keratinocytes and upregulation of LL-37 [120,121]. Tissue-resident memory (TRM) cells are equally important. In the pathological microenvironment of psoriasis, CD8 + TRM cells are increased and involved in disease recurrence or metastasis [122]. In addition, tyrosine kinase 2 (TYK2) can be activated by cytokines such as IL-23, IL-12, and IFN-γ, which in turn transcriptionally activate proteins (STATs) to form dimers and enter the nucleus, regulating the expression of relevant genes. This promotes the differentiation and proliferation of Th17 cells, which then secrete cytokines such as IL-17, recruiting inflammatory cells such as neutrophils, ultimately causing an inflammatory response and hyperproliferation of keratinocytes in the skin.

**Figure 7:**
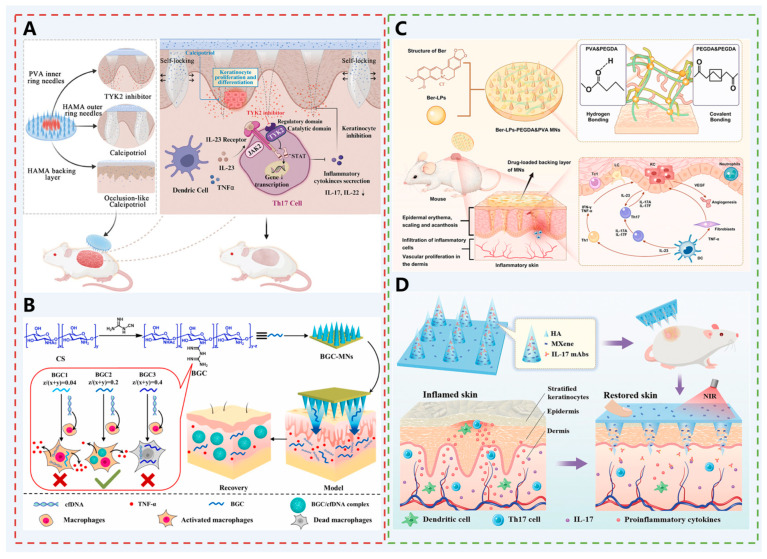
(**A**) Schematic illustration of design and mechanism of self-locking Deu@Cal MN-mediated antiproliferative and immunomodulatory effects for psoriasis therapy. Reprinted with permission from ref. [62].It is an open access article published by John Wiley and Sons. (**B**) Schematic illustration of topical therapy by scavenging cfDNA for psoriasis skin. Reprinted with permission from ref. [63]. Copyright 2024, with permission from Elsevier. (**C**) Liposomes-in-hydrogel MNs loaded with berberine hydrochloride alleviated psoriasis lesions in mice, achieving sustained and controlled drug delivery. Reprinted with permission from ref. [64]. It is an open access article published by Elsevier. (**D**) Schematic design of the application of mAbs-loaded photothermal responsive MN patch for psoriasis treatment. Reprinted with permission from ref. [65]. Copyright 2022, with permission from John Wiley and Sons.

Deucravacitinib (Deu) is a mutational inhibitor of TYK2 and is quite effective in treating psoriasis [123,124]. However, the systemic effects of oral Deu can cause adverse reactions such as upper respiratory tract infections and herpes zoster, and it is contraindicated in patients with severe hepatic impairment or latent tuberculosis infection [125]. Calcipotriol (Cal), a vitamin D derivative, inhibits the proliferation of keratinocytes and regulates the differentiation of keratinocytes, inhibiting the symptoms of localized thickened lesions in some patients with psoriasis. However, its poor skin permeability limits its application. Based on this, Wang et al. developed a dual-release MNs patch [62]. The PVA needle was able to completely penetrate the transdermal barrier and rapidly release Deu in a single dose to modulate the local immune microenvironment and inhibit the spread of skin-derived systemic inflammation. Cal was also loaded into the outer ring and backing layer of the MN patch to resist the sustained proliferation of keratin-forming cells (Figure 7A). In vivo experiments demonstrated that Deu @ Cal MNs effectively down-regulated the psoriasis-associated IL-23/IL-17 pathway and inhibited epidermal hyperplasia, realizing the synergistic effect of immunomodulation and anti-proliferation, which provided a new therapeutic strategy for the treatment of psoriasis.

In addition, it has been shown that the cGAS-STING pathway is highly expressed in psoriasis. This pathway mediates the secretion of cytokines that create a positive feedback loop between skin cells and immune cells, amplifying the inflammatory response. Abnormally elevated cell-free DNA (cfDNA) activates the pathway, leading to an increased inflammatory response in psoriasis. It also stimulates abnormal proliferation of keratin-forming cells (KCs), exacerbating psoriasis development [126]. Cationic polymers can bind to cfDNA through electrostatic interactions to form complexes, creating a spatial site barrier that makes it difficult for cfDNA to bind to receptors on the surface of immune cells, such as TLR9. The complexes may also be taken up by the cell, degraded, or isolated within the cell. The downstream inflammatory signaling pathway cannot be activated, and the inflammatory response can be suppressed. Based on this, Liu et al. designed bicinchoninic chitosan MNs (BGC-MNs) to treat psoriasis by removing cfDNA from the dermis [63]. Chitosan, as a biocompatible polysaccharide, has several applications in drug delivery. It was modified with bisguanidine to prepare bisguanidine chitosan (BGC), a cationic polymer with cfDNA binding ability (Figure 7B). MNs with certain mechanical properties were prepared using BGC as a matrix to overcome the thickened and dense skin barrier under the pathological conditions of psoriasis, and effective treatment was achieved in a mouse model of psoriasis.

Shen et al. innovatively designed and constructed a hydrogel MN (Ber-LPs-PEGDA&PVA MNs) drug delivery system loaded with hydrochloride berberine liposomes for the highly effective treatment of psoriasis [64] (Figure 7C). This system achieves efficient transdermal drug delivery and controlled release by embedding berberine hydrochloride liposomes—characterized by high encapsulation efficiency and excellent stability—into hydrogel MNs prepared via photopolymerization of PVA and PEGDA. The MNs exhibit superior mechanical strength, skin penetration capability, and biocompatibility, while significantly scavenging free radicals and alleviating oxidative stress. In a imiquimod-induced psoriasis mouse model, this MN system effectively alleviated skin lesion symptoms, reduced epidermal thickness, inflammatory cell infiltration, and inflammatory factors (e.g., TNF-α, IL-17), and suppressed the expression of angiogenesis-related proteins (CD31, VEGF). This demonstrates therapeutic potential superior to traditional delivery methods, offering a novel strategy for treating psoriasis and other skin diseases.

Biological agents (such as monoclonal antibodies) are considered the most promising treatment for psoriasis due to their high targeting specificity and efficacy. However, they present significant limitations: systemic administration may induce severe immunosuppression and increase the risk of pathogen infection, while topical formulations fail to effectively penetrate affected skin lesions. To address these challenges, Wu et al. developed a photothermal hyaluronic acid MN patch loaded with the biologic IL-17 monoclonal antibody (IL-17 mAbs) and the two-dimensional biodegradable niobium carbide material Mxene [65] (Figure 7D). Leveraging MXene’s exceptional photothermal conversion properties, near-infrared (NIR) irradiation rapidly converts light energy into heat, causing the HA MN matrix to dissolve quickly. This enables on-demand, rapid release of IL-17 mAbs, thereby inhibiting the IL-17 signaling pathway, suppressing epidermal thickening, and reducing inflammatory cell infiltration and key inflammatory factors (such as TNF-α, IL-23, IL-17). This photothermally responsive MN system enables localized, controlled, and efficient delivery of biologics, offering a novel therapeutic strategy for psoriasis and other inflammatory skin diseases.

Fatma Moawad et al. innovatively developed a detachable-tip MN patch for long-term psoriasis management, enabling sustained release of methotrexate (MTX) and phellin (Ph) [66]. The MN tips, composed of poly(lactic-co-glycolic acid) (PLGA) and poly(lactic acid) (PLA), exhibit excellent mechanical strength and slow degradation properties, ensuring sustained drug release within the skin layers. The body of the MN utilizes rapidly dissolving hyaluronic acid (HA), facilitating swift separation and implantation of the drug-loaded tip after skin penetration. MTX, the gold standard psoriasis treatment, acts by inhibiting cell proliferation and immune responses but carries significant systemic toxicity. Ph, a natural flavonoid compound, exhibits anti-inflammatory, antioxidant, and antiproliferative properties with superior safety. This MN system delivers drug release over several days with a single application, equivalent to two subcutaneous injections, significantly improving patient compliance while reducing systemic toxicity. In vitro and in vivo studies validated its superior mechanical properties, drug release behavior, antiproliferative activity, and therapeutic efficacy in psoriasis models. This innovation offers an efficient, safe, minimally invasive novel delivery strategy for long-term psoriasis management. Its simple fabrication process (no nanocrystal preparation required), low cost, room-temperature storage stability (3 months), and absence of biohazardous waste demonstrate strong clinical translation potential.

### 2.6. MNs to Treat Atopic Dermatitis

Atopic dermatitis (AD) is a common chronic inflammatory skin disease with clinical manifestations of recurrent eczematous lesions and intense itching, which severely affects patients’ mental health and quality of life. Various cells in the skin, such as keratinocytes and Langerhans cells, recognize pathogen-associated molecular patterns (PAMPs) through pattern recognition receptors (PRRs), activating both intrinsic and adaptive immunity [127]. However, patients with AD have abnormal PRRs function, such as impaired TLR2 function, which affects the production of antimicrobial peptides (AMPs) and cytokines, making the skin susceptible to infection and triggering an inflammatory response [128]. In addition, AD lesions are usually colonized by *S. aureus* and secrete superantigens, triggering Th2 cell-associated immune dysregulation and skin inflammation, which further exacerbate epidermal barrier damage [129].

**Figure 8:**
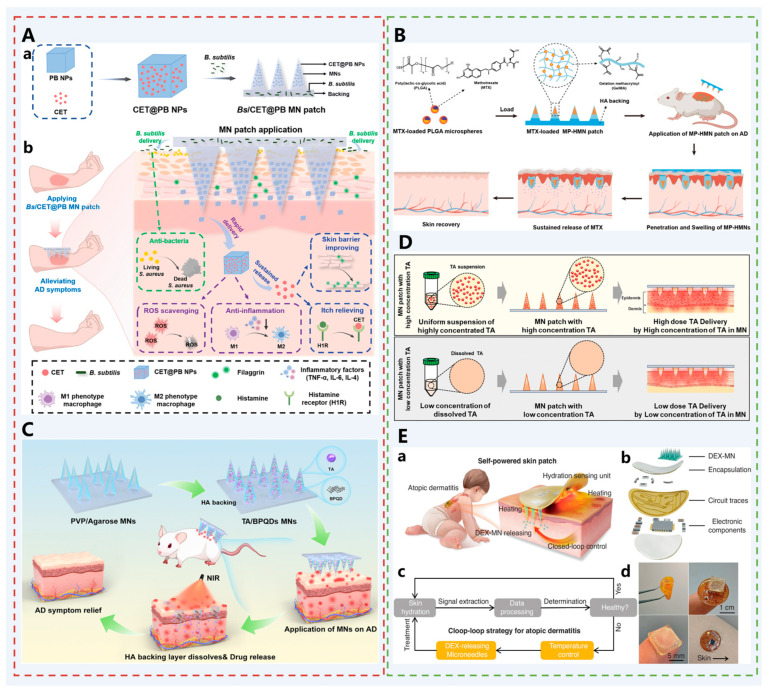
(**A-a**) Schematic illustration of the MN patch composition. (**A-b**) Schematic of the double-layered Bs/CET@PB MN patch for treating AD through anti-bacteria, ROS scavenging, anti-inflammation, itch relieving, and skin barrier improving effects. Reprinted with permission from ref. [67]. Copyright 2025, with permission from John Wiley and Sons. (**B**) Schematic illustration of design and application of MTX-loaded MP-HMN patch for the treatment of AD. Reprinted with permission from ref. [68]. Copyright 2025, with permission from Elsevier. (**C**) Schematic illustration for the preparation of multifunctional TA/BPQD MN patches via template replication method and their therapeutic application on AD-modeled mice. Reprinted with permission from ref. [69]. It is an open access article published byWiley. (**D**)Fabrication of TA–DMN using suspended and dissolved TA–polymer viscous solution and its corresponding TA delivery via DMN. Reprinted with permission from ref. [70]. Copyright 2021, with permission from John Wiley and Sons. (**E-a**) Schematic illustration of the system for atopic dermatitis therapy. (**E-b**) Exploded view of the skin patch. (**E-c**) Workflow diagram of the system operation. (**E-d**) Representative optical images showcasing the skin patch. Reprinted with permission from ref. [71]. It is an open access article published by Nature Publishing Group.

To address the problem of *S. aureus* colonization, Zhang et al. reported a multifunctional bilayer MN patch for long-term AD treatment by integrating therapeutic nanoparticles and live bacteria (Figure 8A). The MNs patch was a water-soluble matrix with a backing layer doped with Bacillus subtilis (*B. subtilis*, Bs), and the tip of the needle encapsulated containing hydrochloride Cetirizine in Prussian Blue NP (CET@PB NP) [67]. Upon administration, the backing layer rapidly dissolved. Bacillus subtilis attached to the skin surface and survived for more than 9 days, competitively inhibiting *S. aureus*, thereby ameliorating the microbial imbalance on the skin surface. In AD, histamine is one of the important mediators of itching and inflammatory response. As an H1 receptor antagonist, CET exerts antiallergic and antipruritic effects by blocking the binding of histamine to the receptor, which in turn reduces the scratching behavior of patients. This can indirectly reduce the aggravation of skin damage and inflammation caused by scratching, which can help repair the skin barrier and ultimately alleviate the AD disease. After implantation into the skin, MNs rapidly dissolved and continuously released CET@PB NP. In addition to reducing itching and repairing the skin barrier, CET@PB NP also had the pharmacological activity of scavenging ROS and reducing the level of inflammatory factors, which can improve the pathological microenvironment of oxidative stress and immune dysregulation in AD. Ultimately, AD was successfully treated with the synergistic effect of scavenging reactive oxygen species, improving the skin barrier, relieving itching, and correcting microbial imbalance.

MTX is a folate antagonist capable of exerting anti-inflammatory effects by inhibiting DNA synthesis and immune cell proliferation, effectively treating AD. However, the systemic effects of oral administration can cause serious adverse effects, including hepatotoxicity, nausea, vomiting, leukopenia, and bone marrow suppression. Moreover, as a chronic disease, repeated doses of MTX are required for AD treatment. To address these issues, Shi et al. developed a microsphere-integrated hydrogel MNs (MP-HMN) patch for the sustained delivery of MTX for the treatment of AD [68] (Figure 8B. MTX was encapsulated in biodegradable polylactic-hydroxyglycolic acid copolymer (PLGA) microspheres, mixed with a GelMA blend. The MP-HMN arrays were prepared by the double-casting micro-molding technique. The backing was made of HA. Upon administration, the HA backing dissolved rapidly upon contact with the skin interstitial fluid. The microspheres conferred additional mechanical properties to the needle body for smooth implantation into the skin. The GelMA hydrogel dissolved gradually after implantation, reducing the abrupt release of MTX and sustaining drug release for up to 12 days. It significantly alleviated AD symptoms, down-regulated the expression of inflammation-related cytokines in an AD mouse model, and achieved better therapeutic effects with a lower dosing frequency.

Topical application or injection of the immunosuppressive steroid triamcinolone acetonide (TA) is a common clinical approach for treating atopic dermatitis (AD). However, chronic AD lesions exhibit thickened skin, making penetration of topical medications challenging. Additionally, TA has extremely low solubility, and conventional MNs cannot carry sufficient doses to meet clinical demands. Therefore, Song et al. designed a near-infrared (NIR)-responsive multifunctional MN patch for treating AD [69] (Figure 8C). The patch comprises a hyaluronic acid (HA) backing layer and needle tips containing black phosphorus quantum dots (BPQDs), low-melting-point agarose, polyvinylpyrrolidone (PVP), and triamcinolone acetonide (TA). The HA backing serves as a long-lasting moisturizer to alleviate AD skin dryness; BPQDs exhibit excellent photothermal conversion properties, generating heat under NIR irradiation to induce phase transition in the agarose gel, thereby enabling controlled TA release; PVP enhances the mechanical strength of the needle tips, ensuring MNs can penetrate the thickened SC of AD patients. Synergistic action of components: PVP guarantees penetration capability, BPQDs enable smart drug delivery, HA provides sustained hydration, and TA exerts anti-inflammatory therapeutic effects. This MN system demonstrated significant efficacy in AD mouse models, reducing epidermal thickening, suppressing inflammatory cell infiltration, and lowering IgE and IL-4 levels, showcasing potential applications in AD treatment and other biomedical fields.

Additionally, enhancing the loading capacity of TA in MNs is an effective approach to improve therapeutic efficacy. Jiang et al. pioneered a TA suspension strategy, significantly reducing TA particle size from 25.1 μm in conventional injections to 5.2 μm via ultrasonic processing [70] (Figure 8D). Combined with optimized polymer composition (polyvinylpyrrolidone PVP and hyaluronic acid HA), this enhanced suspension stability and prevented particle sedimentation. This formulation achieves uniform dispersion of TA particles while maintaining high viscosity, successfully encapsulating 2 mg of TA in each DMN patch containing 108 needles—reaching the dose required for clinical treatment. This provides a novel strategy for transdermal delivery of high-dose steroids.

Although effective delivery of steroids can successfully treat atopic dermatitis, their long-term use may lead to steroid resistance. Considering the clinical manifestations of dry skin and impaired barrier function in atopic dermatitis, Liang et al. proposed a self-powered closed-loop skin patch [71] (Figure 8E). This patch integrates a piezoelectric generator, hydration sensing unit, MN therapy module, and flexible circuitry to achieve a fully self-powered system without external power sources. Its core innovation lies in harvesting mechanical energy from patients’ daily activities and converting it into electrical energy to sustain long-term system operation. The hydration sensing unit monitors skin hydration status in real-time based on changes in skin thermal conductivity, offering high sensitivity and rapid response. Upon detecting abnormal skin hydration persisting for 65 s, the system automatically activates the thermoregulatory MN module. Heating to approximately 42 °C melts the phase-change material, precisely releasing the anti-inflammatory drug sodium phosphate dexamethasone for on-demand therapy. In mouse models, the patch significantly improved epidermal thickness, reduced inflammatory cytokine IL-4 levels, and mitigated splenic lesions, demonstrating excellent therapeutic efficacy and biocompatibility. The integrated system combines hydration monitoring, drug delivery, and energy harvesting, enabling fully closed-loop control from sensing to treatment without human intervention. This approach offers a novel strategy for intelligent management of chronic skin diseases and advances the development of self-powered biomedical engineering.

### 2.7. MNs to Treat Vitiligo

Vitiligo is an autoimmune skin disease characterized by loss of skin pigmentation to form white patches, which has a serious negative impact on patients’ quality of life. Damage to melanocytes and reduced endogenous melanin synthesis are the direct causes of reduced melanin levels [130], and oxidative stress and autoimmune responses are important in the progression of vitiligo. Under oxidative stress, apoptotic melanocytes are exposed to self-antigens and release high mobility group protein 1 (HMGB1), which triggers autoimmune activation and recruitment of CD8+ T cells [131,132]. These CD8+ T cells further attack surrounding melanocytes, leading to melanocyte damage and ROS accumulation [133,134,135], which in turn exacerbates oxidative stress and creating a vicious cycle. In addition, accumulated CD8+ T cells release IFN-γ to activate the JAK-STAT signaling pathway in keratinocytes, which induces keratinocytes to express and release T-cell chemokines, exacerbating the vitiligo disease process.

**Figure 9:**
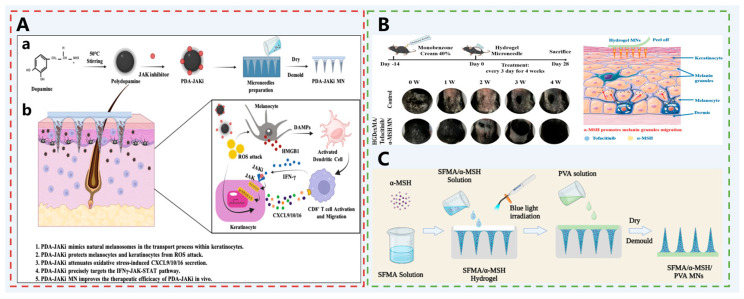
(**A-a**) The synthesis process of PDA-JAKi MN. (**A-b**) The mechanism mediated by PDA-JAKi MN Reprinted with permission from ref. [72]. It is an open access article published by BioMed Central. (**B**) Schematic diagram and animal experiment Results of HGDexMA MNs therapy for vitiligo. Reprinted with permission from ref. [73]. Copyright 2023, with permission from Elsevier. (**C**) Schematic illustration of design and application of α-MSH-based hydrogel MNs for the treatment of vitiligo. Reprinted with permission from ref. [74]. Copyright 2023, with permission from American Chemical Society.

Polydopamine (PDA) is an artificial melanin-like nanoparticle with ROS scavenging ability that mimics the behavior of natural melanosomes in terms of transport, distribution, and uptake [136]. Tofacitinib, a Janus kinase inhibitor (JAKi), can target the IFN-γ-JAK-STAT signaling pathway and effectively inhibit the activation of CD8+ T cells, and reverse the inflammatory microenvironment. Based on this, Li et al. reported a novel agent for vitiligo treatment [72]. PDA nanoparticles were loaded with tofacitinib (PDA-JAKi), which was precisely delivered at the lesion site with the help of HA MNs (Figure 9A). PDA-JAKi was able to remove excessive ROS in the vitiligo pathological microenvironment effectively, maintain the integrity of the mitochondria, and inhibit the release of HMGB1, which protects melanocytes and keratinocytes from apoptosis. It also inhibited the JAK-STAT pathway in keratinocytes and reduced the expression of phospho-STAT 1 (p-STAT 1) and phospho-STAT 3 (p-STAT 3) as well as the secretion of related chemokines, which demonstrated good therapeutic effects in animal models: the level of ROS and the infiltration of CD8+ + T cells were significantly reduced, and the melanocyte number and melanin content, symptom reduction, and restoration of pigmentation in the affected areas.

Similarly, Liang et al. designed a dual-crosslinked hydrogel MN system based on dextran methacrylate (DexMA) and cyclodextrin–diamantane host–guest supramolecular (HGSM) for the synergistic delivery of the JAK inhibitor tofacitinib and the melanocyte-protective agent α-MSH to treat vitiligo [73] (Figure 9B). DexMA provides excellent biocompatibility and mechanical strength, while HGSM further enhances the hydrogel’s network density and mechanical properties through host–guest assembly, enabling effective penetration of the stratum corneum. Tofacitinib blocks the immune positive feedback loop by inhibiting the JAK pathway, reducing CD8^+^ T cell-mediated inflammation; α-MSH promotes melanin synthesis and migration by activating the melanocortin receptor 1 (MC1R). This MN system enables sustained-release drug delivery at the target site, significantly promoting epidermal and follicular melanin deposition. In animal models, it demonstrates superior repigmentation effects compared to conventional drugs, offering a novel, highly effective, and safe transdermal delivery strategy for vitiligo treatment.

Aiming at the direct cause of melanocyte damage and reduced endogenous melanin synthesis, reducing melanocyte damage and promoting melanin synthesis from the source is an effective path to treat vitiligo. Melanocyte-stimulating hormone (α-MSH) is a neuroendocrine tripeptide hormone. It activates adenylate cyclase (AC) by binding to the melanocortin 1 receptor (MC1R) on melanocytes, upregulating intracellular cyclic adenosine monophosphate (cAMP) expression. cAMP triggers protein kinase A (PKA) to activate TYR, which utilizes the superoxide anion as a substrate for the production of melanin and reduces the level of intracellular peroxides while promoting melanin production to protect the melanocytes from oxidative stress [137,138]. In addition, α-MSH has a role in the regulation of inflammation, inhibiting the activation of pro-inflammatory transcription factor nuclear factor NF-κB induced by inflammatory factors such as TNF-α, IL-1, and others [139]. Therefore, Chong et al. developed α-MSH-based hydrogel MNs [74] (Figure 9C). Photocrosslinked methacrylate filipin protein (SFMA) was selected as the matrix to prepare MNs with good biocompatibility and a certain mechanical strength. The tip was loaded with α-MSH, which was delivered directly to the epidermis to perform biological functions, thus accelerating the synthesis and transport of melanin, promoting the repigmentation of the epidermis, and ultimately effectively treating vitiligo.

## 3. Conclusions and Perspective

As a revolutionary breakthrough in transdermal drug delivery, MN technology demonstrates unique application value in dermatological treatment. Addressing the inherent issues of low bioavailability caused by gastrointestinal digestion and hepatic first-pass metabolism in oral administration, MNs enable drug delivery through skin or mucosal membranes with minimal systemic exposure [20]. Addressing the pain and bleeding associated with injections, MNs bypass the nerve-rich dermis due to their minute size, resulting in virtually zero pain and bleeding and significantly improving patient compliance [10]. Overcoming the limitation of traditional transdermal patches to effectively deliver only lipophilic small molecules, MNs mechanically breach the stratum corneum barrier to efficiently deliver diverse therapeutic agents, including vaccines [140], biologics [141], and nanoparticles [142]. This review summarizes recent MN technology advancements in treating androgenetic alopecia, acne, scars, melanoma, psoriasis, atopic dermatitis, and vitiligo. Different MN types combine the aforementioned advantages of high efficiency, minimal invasiveness, painlessness, and high patient compliance. They can also synergize with photothermal therapy [47], immunomodulation [62], and other strategies to significantly enhance therapeutic outcomes.

To understand the current clinical development status of MNs (MNs), we searched ClinicalTrials.gov using the keyword “MN.” A total of 152 MN-related clinical trials were identified, of which 19.73% (30/152) were Phase I trials, 19.73% (30/57) were Phase II trials, 9.87% (15/152) were Phase III trials, and 2.63% (4/152) were Phase IV trials. The majority of trials (46.7%, 71/152) were not applicable (NA). Commercially, MNs applications remain heavily skewed toward esthetics, with over 80% of products targeting anti-aging, acne treatment, and scar remodeling, while offerings for chronic skin conditions remain scarce [143]. Therefore, we conclude that while MN technology demonstrates transformative potential for medical practice due to its painless, efficient, and patient-friendly delivery method, the path to widespread clinical adoption remains fraught with numerous challenges requiring systematic resolution. These complex challenges span multiple dimensions, including technological maturity, regulatory approval, manufacturing processes, and end-user applications.

At the technical level, the core challenge for intelligent responsive MNs lies in achieving a unified balance of high integration, intelligence, and reliability. Most current so-called “smart” MN devices possess relatively limited functionality, typically combining only basic physical or chemical sensors to monitor a finite set of biomarkers and trigger simple drug release mechanisms [144]. This rudimentary level of intelligence struggles to address the complexities of real-world clinical settings. Factors such as biological fouling, significant inter-individual variations in skin characteristics, and dynamically changing physiological states can severely compromise their performance. The response logic of these systems is often based on pre-set thresholds, lacking real-time data analysis and adaptive decision-making capabilities powered by artificial intelligence (AI) and machine learning (ML) [145]. This limitation restricts their application in scenarios requiring complex feedback regulation, such as chronic disease management. Furthermore, miniaturizing and seamlessly integrating microsensors, microprocessors, and wireless communication modules into MN arrays without compromising mechanical strength, wear comfort, and biocompatibility presents a formidable multidisciplinary engineering challenge [146]. The solution lies in deeply integrating materials science, electronic engineering, and data science. Future developments must focus on creating more advanced, stable, and contamination-resistant biosensors with embedded edge computing capabilities to enable preliminary data processing. By introducing AI algorithms for real-time analysis of massive continuous monitoring data—either in the cloud or locally—predictive models can be built to deliver truly personalized adaptive therapy [147,148]. For instance, insulin release rates could be dynamically adjusted based on projected blood glucose trends, rather than merely reacting to current hyperglycemia [149].

The regulatory framework represents a significant barrier for MN technology transitioning from the laboratory to the market, with its constraints being particularly pronounced. Agencies such as the U.S. Food and Drug Administration (FDA) and the European Medicines Agency (EMA) classify MN products as medical devices, combination products (device + drug/biological), or new drugs, with complex and stringent approval pathways [140]. For MNs integrated with smart systems, regulatory thresholds are even higher, as they must simultaneously demonstrate the safety and efficacy of the device component alongside the pharmacokinetics, pharmacodynamics, and stability of the drug component. Regulators demand comprehensive preclinical data spanning material biocompatibility, in vivo/in vitro degradation behavior, drug release kinetics, mechanical reliability, and mass production quality consistency. Subsequently, rigorous phased clinical trials (Phases I–IV) must validate safety and efficacy across diverse populations—a lengthy and prohibitively costly process [150]. Currently, most MN clinical trials remain in early stages, with indications concentrated in esthetic medicine [151]. This reflects insufficient evidence supporting their transition to high-value medical applications. To overcome this limitation, industry and regulators must initiate close dialog at an early stage. Promoting regulatory coordination and mutual recognition (e.g., agreements between the FDA and EMA) is crucial to avoid duplicative approvals and accelerate global market access. Simultaneously, establishing dedicated industry standards for MN technology—such as mechanical strength testing, sterility assurance, and drug release efficiency—will provide clear, unified guidelines for product development and review, reducing uncertainty [152]. Generating real-world evidence (RWE) to supplement traditional clinical trial data will also help continuously demonstrate long-term safety and efficacy, supporting clinical translation and expanded indications.

At the manufacturing level, regionally speaking, North American companies dominate high-end medical devices, while Asian manufacturers capture global markets through cost-effective mass production (20 million units/year) and competitive pricing (30% lower than Western counterparts). Currently, 3D printing technology is fundamentally reshaping the development and mass production models for MNs, offering revolutionary solutions to overcome traditional manufacturing bottlenecks [151]. Conventional microfabrication processes, such as micro-molding, face limitations in achieving complex geometries, personalized customization, and rapid iteration, coupled with high initial mold costs and inflexible adjustments [153]. 3D printing, particularly high-resolution techniques (e.g., two-photon polymerization achieving < 1 μm precision [154]), enables researchers to directly “print” MN arrays featuring intricate internal channels, multi-level structures, irregular tips, and customized dimensions based on digital models [155]. This digital manufacturing approach dramatically accelerates prototyping and optimization cycles, facilitating tailored designs for specific applications like irregular wound dressings [156]. Regarding mass production, while high-speed, large-volume 3D printing remains challenging, rapid progress is being made. High-speed photopolymerization techniques like multi-nozzle array printing [157] and Continuous Liquid Interface Production (CLIP) [158]are focused on increasing printing throughput. Through digital workflows, the same production platform can rapidly switch between manufacturing different MN designs without costly mold changes [159], enabling “mass customization.” This undoubtedly lays a solid foundation for MNs to address diverse clinical needs and achieve economically viable large-scale applications.

At the end-user level, ensuring patients can correctly and safely self-administer MN devices is a prerequisite for their widespread adoption in home settings, urgently requiring the integration of reliable verification systems. Improper application—such as insufficient pressure, incorrect angle, inadequate duration, or inappropriate site—may result in suboptimal drug delivery, diminished efficacy, or even skin damage [160]. Currently, most MN patches rely on user self-monitoring, lacking objective feedback mechanisms. The solution lies in directly integrating intelligent verification systems into the delivery devices. This could include simple mechanical or electronic feedback mechanisms. For example, the device could be designed to emit a visual (LED color change) or auditory (beep) signal only after sufficient and uniform pressure is applied for a predetermined duration, confirming successful drug release. More advanced systems could integrate thin-film pressure sensors and inertial measurement units (IMUs) to monitor pressure distribution, angle, and micro-movements during application, ensuring proper technique. This data could even be transmitted via Bluetooth to a smartphone app, providing users with guided feedback and recording compliance history, while offering healthcare providers a means for remote monitoring. Furthermore, device design should inherently follow “foolproof” principles, making incorrect application physically difficult. This integrated hardware-software verification system significantly reduces user variability, safeguards treatment efficacy, and builds confidence among patients and healthcare professionals in home MN therapy—ultimately driving its widespread adoption.

In summary, the journey of MN technology from the laboratory to clinical application is far from a simple technological iteration; it represents a profound multidimensional transformation. Four formidable barriers stand in the way of its large-scale translation: the technical complexity of intelligent system integration, the rigor and uncertainty of regulatory frameworks, the economic bottlenecks of mass manufacturing, and the reliability assurance for end-user applications. This demands we transcend the limitations of solitary, single-discipline efforts and instead initiate a cross-disciplinary “grand convergence”—fusing the wisdom of materials science, electronic engineering, data science, and clinical medicine to disrupt traditional R&D paradigms through deep interdisciplinary integration; Drive innovation in regulatory science to establish agile, transparent, and mutually recognized approval pathways; achieve revolutionary breakthroughs in manufacturing technology to enable high-quality personalized production with both scale and cost advantages; and uphold user-centric design principles, embedding safety and usability into the very soul of the product. Only through such systematic thinking and boldness can these challenges be overcome, transforming MN technology’s immense potential from blueprint to reality. This will finally bridge the last mile from “micron-scale needles” to “macro-scale applications,” unleashing its colossal power to pioneer a new era of precision medicine.

## Figures and Tables

**Table 2:** Overview of MNs for Treating Various Skin Diseases.

Diseases	MNs’ Names	Drug-Loading Agents and Their Corresponding Functions	References
AGA	CS-F-MN	FIN-NCs: Serves as the main drug to inhibit 5α-reductase and reduce follicle miniaturization. CS: Coats the nanocrystals to enhance positive charge for improved follicular targeting and accumulation.	[43]
	CeMN	Possesses CAT and SOD activities, alleviates oxidative stress in the pathological microenvironment of AGA, protects dermal papilla cells.	[27]
	Se-HEVs-AMN	SeNPs: Antioxidant and anti-androgenic; HEVs: Hypoxia pretreatment upregulates HIF1α, promotes VEGF transcription and angiogenesis; APS: Activates the Nrf2/HO-1 pathway, inhibits inflammation and induces M2 macrophage polarization, synergistically promoting hair growth.	[44]
	V-R-MN	Ritlecitinib: inhibits the attack of hair follicle cells by CD8+ T cells and natural killer cells, and regulates the perifollicular immune microenvironment; VEGF: Improves the perifollicular vascular microenvironment, and provides nutrients for hair follicles.	[45]
	PQFN MN	PQFN: Scavenges ROS, promotes angiogenesis, and reverses DHT-induced senescence of dermal papilla cells; NaHCO_3_ and Citric Acid: React with skin interstitial fluid to generate CO_2_, driving PQFN into the deeper dermis.	[46]
Acne	ZIF-8-ICG @ MNs	ICG: As a photosensitizer, generates ROS under NIR laser activation, inhibits *P. acnes* proliferation and reduces inflammatory factor expression; ZIF-8: A pH-responsive carrier that degrades in the acidic microenvironment of bacterial infection, releases Zn^2+^ to destroy bacterial cell membranes, while avoiding aggregation-induced quenching of ICG and improving its photostability.	[47]
	E @ P-EO-HA MNs	EO: Antibacterial and anti-inflammatory effects, promoting the repair of *P. acnes*-infected skin; PDA NP: As a photothermal agent, rapidly generates heat under 808 nm NIR laser irradiation, destroys sebaceous glands and inhibits *P. acnes* proliferation.	[48]
	EGCG @ BSP/HA MNs	EGCG: Exerts broad-spectrum antibacterial, anti-inflammatory, and antioxidant effects; BSP and HA: Constitute the needle body, with anti-inflammatory, antioxidant, and wound-healing promoting functions; PVA and DE: Constitute the base, and the porous structure of DE absorbs acne pus, prevents bacterial reinfection, and provides a clean environment for drug action.	[49]
	ZnTCPP@ZnO MN	ZnTCPP@ZnO: Ultrasound-responsive nanocomposites that promote oxygen activation and generate singlet oxygen (^1^O_2_) under ultrasound, achieving a 99.73% clearance rate of P. acnes; Zn^2+^: Released from the composites, upregulates metallothionein and DNA replication-related genes, and promotes cell proliferation and skin repair.	[50]
	Multifunctional nanocomposites MNs	CS: Positively charged, efficiently captures *P. acnes*; CUR: Has fluorescent properties, enabling rapid visual detection of bacterial concentration; TSIIA: Exerts anti-inflammatory effects, and combined with PDT, matches personalized light doses according to fluorescence intensity to accurately eliminate bacteria and alleviate inflammation.	[51]
Scar	BSP-MNs-QUE @ HSF/CDF	QUE: Regulates the Wnt/β-catenin and JAK2/STAT3 signaling pathways, and reduces the expression of type I and type III collagen in hypertrophic scars; CDF: A drug carrier with high drug loading capacity and good biocompatibility; BSP: Constitutes the MNs matrix, with anti-inflammatory, antioxidant, and wound-healing promoting functions. HSF Membrane: Enables QUE@HSF/CDF to specifically target HSF.	[52]
	CAD NPs MNs	AuNCs: Promote ROS production, consume GSH, and induce cell apoptosis; DHA: Generates free radicals, triggers oxidative stress and induces iron deficiency; CB[n]s: Self-assembles with AuNCs and DHA into pH-responsive CAD NPs, mediating ferroptosis–apoptosis combined effects to inhibit excessive proliferation of scar fibroblasts.	[53]
	OUSMN	UCNPs: Surface-modified with HKN15 and RB, emit red light for real-time imaging to monitor the targeting process under 990 nm laser excitation, generate ^1^O_2_ under 808 nm laser excitation, degrade ferritin highly expressed in KFs, release iron ions to induce endogenous ferroptosis, and simultaneously synergize with PDT to inhibit the PI3K-AKT and mTOR pathways.	[54]
	MN-C/P-Z	CUR: Continuously inhibits the expression of α-SMA and type I collagen, prevents fibroblasts from differentiating into myofibroblasts, and reduces excessive collagen deposition; ZnO NPs: Constitute the MNs base, form a physical barrier, inhibit Staphylococcus aureus and Escherichia coli, prevent infection and create a moist healing environment;	[55]
	HFSVM	Verteporfin: A YAP inhibitor that inhibits the YAP signaling pathway, reduces the transformation of En-1-positive fibroblasts, and inhibits scar formation; SDA: Consumes ROS and decomposes to produce oxygen under high ROS conditions, alleviates tissue hypoxia, and activates the ERK1/2 and HO-1 pathways to promote angiogenesis; HA-FPBA: Crosslinks with SDA via borate ester bonds to form MNs, and the borate ester bonds dissociate under high glucose conditions to control the release of Verteporfin.	[56]
Melanoma	Groove MN patch	CCL22: Modified on the surface of MNs, acts as a chemoattractant to divert Treg from the TME, increases the ratio of CD8+ T cells/Treg cells, and enhances the inhibitory effect of TCR T cells or CAR T cells on melanoma in ACT.	[57]
	PcNP/TRA-HA-Tyr/CLG-MN	Pc: As a photosensitizer, exerts PDT effects under NIR irradiation to kill tumor cells; TRA: Targets the MEK pathway and inhibits melanoma growth and proliferation; CLG: degrades collagen in the tumor ECM, and improves drug diffusion and distribution in tumors.	[58]
	DHA @ HPFe-MN	DHA: Catalyzed by Fe^2+^ generated from the reduction of PA-Fe^3+^ in the acidic tumor microenvironment, produces a large amount of ROS to achieve oxygen-independent chemotherapy; HA-ADH-PpIX: A pH-responsive conjugate that degrades to release PpIX in acidic environments, exerting PDT effects under NIR irradiation to further amplify oxidative stress; PA-Fe^3+^: Serves as an “iron reservoir” to provide Fe^3+^ for participating in the catalytic reaction of DHA.	[59]
	D/I @ PATC MN	PATC: Has AIE property to realize visual monitoring of drug release process; also has light-controlled pulsed drug release capability, and a single administration can trigger multiple drug releases through external light irradiation, combining chemotherapy and photothermal therapy to enhance the therapeutic effect on melanoma.	[60]
	Au @ Ag-Pt NPs MN	Au@Ag-Pt NPs: Surface-modified with dopamine, combined with MNs via reversible borate bonds; in the presence of TYR, dopamine is oxidized to benzoquinone, inhibiting the combination of nanoparticles and MNs, resulting in a dual-mode response of SERS signal attenuation and colorimetric signal enhancement; used for early detection and monitoring of melanoma.	[61]
Psoriasis	Deu @ Cal MN	Deu: A TYK2 mutation inhibitor that is rapidly released to regulate the local immune microenvironment, inhibit the spread of skin-derived systemic inflammation, and downregulate the psoriasis-related IL-23/IL-17 pathway; Cal: A vitamin D derivative that is slowly released to inhibit keratinocyte proliferation and regulate keratinocyte differentiation.	[62]
	BGC-MN	BGC: A cationic polymer that binds cfDNA in the dermis through electrostatic interaction, preventing cfDNA from activating the cGAS-STING pathway, and inhibiting inflammatory response and abnormal proliferation of keratinocytes.	[63]
	Ber-LPs-PEGDA and PVA MN	Ber: Has anti-inflammatory and antioxidant effects, inhibits the expression of inflammatory factors, and downregulates angiogenesis-related proteins; LPs: Improve the encapsulation efficiency and stability of Ber, achieving efficient transdermal drug delivery and controlled release.	[64]
	Mxene-MN	IL-17 mAbs: Inhibits the IL-17 signaling pathway, inhibits epidermal thickening, and reduces inflammatory cell infiltration and inflammatory factors; Mxene: Has excellent photothermal conversion properties, generates heat under NIR irradiation to dissolve the HA matrix, triggering on-demand release of IL-17 mAbs.	[65]
	MTX and Ph-loaded PLGA-tipped MNs	MTX: Inhibits cell proliferation and immune response, a “gold standard” drug for psoriasis treatment; Ph: A natural flavonoid compound with anti-inflammatory, antioxidant, and antiproliferative effects and high safety.	[66]
Atopic dermatitis	*Bs*/CET @ BP MN patch	CET: An H1 receptor antagonist that blocks the binding of histamine to receptors, relieves itching, and reduces skin damage and inflammation caused by scratching; PB NP: Scavenges ROS, reduces the level of inflammatory factors, and improves the pathological microenvironment of oxidative stress and immune disorders in AD; Bs: Survives on the skin surface and competitively inhibits *S. aureus*, improving skin microbial imbalance.	[67]
	TA/BPQDS MNs	MTX: Inhibits DNA synthesis and immune cell proliferation, exerting anti-inflammatory effects; PLGA: Encapsulates MTX into microspheres to achieve sustained drug release for up to 12 days, reducing administration frequency.	[68]
	MP-HMN	TA: A glucocorticoid that exerts anti-inflammatory effects for AD treatment; BPQDs: Generates heat under NIR irradiation, inducing agarose phase transition to control TA release;	[69]
	TA-DMN	TA: Anti-inflammatory effect for AD treatment; PVP and HA: Optimize polymer composition, and combine with ultrasonic treatment to reduce the TA particle size from 25.1 μm to 5.2 μm, enhance suspension stability, avoid particle sedimentation, and achieve encapsulation of 2 mg TA per MN patch (containing 108 needles) to reach the clinical therapeutic dose.	[70]
	Self-powered closed-loop skin patch	Dexamethasone Sodium Phosphate: An anti-inflammatory drug that, when the skin hydration signal is abnormal (lasting 65 s), the piezoelectric generator generates heat to melt the phase-change material, achieving on-demand release; Piezoelectric Generator: Collects mechanical energy from human daily activities and converts it into electrical energy to power the system; Hydration Sensing Unit: Monitors skin hydration status in real-time based on changes in skin thermal conductivity to trigger drug release;	[71]
Vitiligo	PDA-JAKi MN	PDA: Melanin-like nanoparticles that scavenge ROS, protect the mitochondrial integrity of melanocytes, inhibit the release of HMGB1, and prevent melanocyte apoptosis; JAKi: Inhibits the IFN-γ-JAK-STAT signaling pathway, reduces CD8+ T cell activation and infiltration, and decreases the expression of inflammatory factors and chemokines;	[72]
	HGDexMA, hydrogel MNs	JAKi: Inhibits the JAK pathway, and reduces CD8+ T cell-mediated inflammation; α-MSH: Activates the MC1R to promote melanin synthesis and migration; DexMA: Provides biocompatibility and mechanical strength; HGSM: Enhances the hydrogel network density and mechanical properties.	[73]
	SFMA/α-MSH/PVA MNs	α-MSH: Activates the MC1R on melanocytes, upregulates intracellular cAMP, activates Tyr, promotes melanin synthesis and transport, and simultaneously inhibits NF-κB activation induced by inflammatory factors; SFMA: Constitutes the MN matrix with good biocompatibility and mechanical strength; PVA: Assists in forming MNs and enhances stability.	[74]

## Data Availability

No new data were created or analyzed in this study. Data sharing is not applicable to this article.

## Abbreviations

The following abbreviations are used in this manuscript:

MNs	Microneedles	SC	Stratum Corneum
TDDS	Transdermal Drug Delivery System	AGA	Androgenetic Alopecia
FIN-NP	Finasteride nanocrystals	CS	Cationic chitosan
CeNZ	Cerium dioxide nano-enzyme	CAT	Catalase
SOD	Superoxide Dismutase	SeNPs	Selenium nanozymes
HEVs	Hypoxia-pretreated extracellular vesicles	APS	Astragalus polysaccharide
HIF1α	Hypoxia-inducible factor 1α	VEGF	Vascular Endothelial Growth Factor
PQFN	Puerarin/quercetin iron-chelating nanoparticles	aPDT	Antimicrobial Photodynamic Therapy
ICG	Indocyanine Green	ZIF-8	Zeolitic Imidazolate Framework-8
2-MeIM	2-Methylimidazole	PDA NP	Polydopamine Nanoparticles
EO	Eugenol	BSP	Bletilla striata polysaccharide
EGCG	Epigallocatechin gallate	PVA	Polyvinyl Alcohol
DE	Diatomaceous Earth	ZnTCPP@ZnO	Zinc tetra(4-carboxyphenyl)porphyrin@Zinc Oxide
CS	Chitosan	CUR	Curcumin
TSIIA	Tanshinone IIA	HSF	Hypertrophic Scar Fibroblasts
CDF	Cyclodextrin metal–organic Framework	QUE	Quercetin
AuNCs	Gold Nanoclusters	DHA	Dihydroartemisinin
CB[n]s	Cucurbiturils	CAD NPs	Cucurbituril-Assembled Drug Nanoparticles
OUSMNs	Orthogonally Upconverting Supramolecular MNs	UCNPs	Upconversion Nanoparticles
RB	Rose Bengal	KFs	Keloid Fibroblasts
PDT	Photodynamic Therapy	PLGA	Poly(lactic-co-glycolic acid)
ZnO NPs	Zinc Oxide Nanoparticles	SDA	Dopamine-functionalized Sericin
HA-FPBA	Hyaluronic Acid-Fluorophenylboronic Acid	ACT	Adoptive T Cell Therapy
TCR	T Cell Receptor	CAR	Chimeric Antigen Receptor
TME	Tumor Microenvironment	Treg	Regulatory T cells
CCL22	Chemokine (C-C motif) ligand 22	CLG	Collagenase
Pc	Photosensitizer	TRA	Trametinib
HA-Tyr	Hyaluronic Acid-Tyramine	PpIX	Protoporphyrin IX
PA-Fe^3+^	Phenanthroline-Fe^3+^ complex	PATC	Thermosensitive polymer with AIE property
AIE	Aggregation-Induced Emission	SERS	Surface-Enhanced Raman Scattering
TYR	Tyrosinase	Deu	Deucravacitinib
Cal	Calcipotriol	cfDNA	Cell-free DNA
BGC	Biguanide-modified Chitosan	Ber	Berberine
LPs	Liposomes	PEGDA	Poly(ethylene glycol) diacrylate
IL-17 mAbs	Interleukin-17 monoclonal antibodies	MXene	Two-dimensional niobium carbide
MTX	Methotrexate	Ph	Phellin
PLA	Polylactic Acid	AD	Atopic Dermatitis
CET	Cetirizine	PB NP	Prussian Blue Nanoparticles
Bs	Bacillus subtilis	TA	Triamcinolone Acetonide
BPQDs	Black Phosphorus Quantum Dots	PDA	Polydopamine
JAKi	Janus Kinase inhibitor	HMGB1	High Mobility Group Box 1
DexMA	Dextran Methacrylate	HGSM	Host–Guest Supramolecular
α-MSH	Alpha-Melanocyte Stimulating Hormone	MC1R	Melanocortin 1 Receptor
SFMA	Silk Fibroin Methacrylate	AC	Adenylate Cyclase
cAMP	Cyclic Adenosine Monophosphate	PKA	Protein Kinase A
NF-κB	Nuclear Factor kappa B		
